# Metabolic, Hormonal, Immunologic, and Genetic Factors Associated With the Incidence of Thyroid Disorders in Polycystic Ovarian Syndrome Patients

**DOI:** 10.7759/cureus.11681

**Published:** 2020-11-24

**Authors:** Jaya Singh, Hilda Wong, Nancy Ahluwalia, Ryan M Go, Michelle A Guerrero-Go

**Affiliations:** 1 Primary Care, California Institute of Behavioral Neurosciences & Psychology, Fairfield, USA; 2 Medicine and Research, Avalon University School of Medicine, Curcaco, CUW; 3 Primary Care, Avalon University School of Medicine, Curcaco, CUW; 4 Primary Care, Medical Univeristy of Silesia, Katowice, POL; 5 Medicine and Surgery, University of Santo Tomas, Manila, PHL

**Keywords:** pcos, thyroid, autoimmune, polycystic ovarian syndrome, thyroiditis, hypothyroidism, hashimoto

## Abstract

Many endocrinopathies have been increasingly affecting females of reproductive age. Polycystic ovary syndrome (PCOS) and hypothyroidism are some of the most common endocrinopathies seen in females. The aim of this study is to find a relationship between the incidence of thyroiditis in polycystic ovarian syndrome patients. Literature review search was conducted via a series of systematic searches using multiple databases which lead to cross-referencing of articles within assigned criteria. Our study resulted in a review of 42 articles pertaining to our study. In this literature review article, factors were identified associating PCOS with thyroiditis. A viable relationship was found between the incidence of PCOS and thyroiditis. The combination of factors seen in this study proposes that clinicians may need to focus on certain markers regarding the screening of the incidence of thyroiditis. We also recommend future cohort studies to be conducted to further confirm the associations identified in this article.

## Introduction and background

Polycystic ovary syndrome (PCOS) is known to be one of the most common reproductive endocrinopathies in females, with a reported prevalence of 3-15% depending on the studied population and the applied diagnostic criteria [1]. Many use the guidelines recommended by the Rotterdam criteria for diagnosis, which mandate the presence of two of the following three clinical findings--hyperandrogenism, ovulatory dysfunction, i.e., oligo-ovulation or anovulation, and polycystic ovaries--plus the exclusion of other diagnoses that could cause hyperandrogenism or ovulatory dysfunction [2]. In addition, PCOS patients may also develop other related endocrine and metabolic diseases, which may lead to increased risk for endometrial cancer, obesity, diabetes mellitus, hypertension, dyslipidemia, many of which may contribute to cardiovascular diseases [3,4]. Lastly, PCOS is known to be one of the most common causes of anovulatory infertility in women of reproductive age. Requiring some women to go through the process of ovulation induction or assisted reproductive technology to become pregnant [5]. 

The exact etiology of PCOS is still unclear and/or not fully understood, but studies suggest either a genetic or metabolic component for the origin of PCOS [3,6]. There is evidence from a twin-family study that indicates a strong genetic component causing PCOS [6]. Many studies over the years have shown a higher presence of autoimmune thyroiditis (AIT) in specifically polycystic ovary syndrome patients [7,8]. Studies show AIT having a prevalence rate of about 18-40% in women with PCOS, depending on PCOS diagnostic criteria and ethnicity [9]. 

AIT is one of the most frequent causes of hypothyroidism in young women living in iodine sufficient regions [10]. Autoimmune thyroiditis also called Hashimoto's thyroiditis (HT), or chronic lymphocytic thyroiditis (CLT), is the most prevalent autoimmune disease that affects up to 5-20% of females in their reproductive years leading to chronic inflammation of the thyroid, with the eventual development of full-blown hypothyroidism [10]. Most patients with thyroiditis are usually positive for antibodies such as thyroid peroxidase (TPO) and/or thyroglobulin (Tg) antibodies. However, many women may have detectable levels of antibodies in the upper limit of normal observed for many years without noticeable thyroid dysfunction, this phenomenon is known as subclinical hypothyroidism (SCH). Often ignored, these patients later in life will develop AIT [11]. 

Many women with PCOS and AIT share many similar clinical findings, such as menstrual irregularities, infertility, obesity, insulin resistance, and dyslipidemia [12,13]. Some studies have also indicated evidence between thyroid autoimmunity and pregnancy outcomes such as miscarriages and preterm birth [14]. Many of these shared findings may be further aggravated as a result of the co-existence of PCOS and AIT, which may be more the reason to further test the associations between PCOS and thyroiditis. Findings of associations may further help in future preventative screenings and in improving the management of care. Without proper knowledge of associated linkage, patients may go undiagnosed hastily and receive improper care that may have been necessary for issues, such as infertility, menstrual abnormalities, uncontrollable diabetes, and many other shared similar clinical findings. The aim of this study is to help further analyze, summarize, and update the information from previous studies done to date on the associations between these disorders.

## Review

Methods

The design of this literature review is a compilation of articles relevant to our topic from the inception of PCOS and thyroiditis till January 2019. Keywords used to search articles were: polycystic ovarian syndrome thyroiditis, polycystic ovarian syndrome autoimmune, autoimmune thyroiditis, Hashimoto's thyroiditis, subclinical hypothyroidism, antithyroid peroxidase antibodies. The data was sourced via PubMed, MEDLINE, PubMed Central, and Google Scholar. Cross-referencing of source material led to the additional articles referenced. The study selection included articles peer-reviewed and met an acceptable level of quality. The following exclusion/inclusion criteria were used to source articles: age -- from Inception to 2019; type of study -- all types; peer-reviewed -- yes; gray literature -- no; population -- all; language -- all; country -- all. 

Discussion

Endocrine abnormalities remain a top priority as they are still widely prevalent in women of reproductive age. From the research done in this present literature review, it has been established that PCOS and thyroiditis have a positive correlation according to the majority but one study found. Further research and clinical trials are being conducted to further explain direct associations between these conditions. However, many studies to date have already concluded the associations of hormonal, metabolic, genetic, and immunologic factors that may be a potential connection to the increased risk of thyroiditis in PCOS patients. 

Metabolic Factors

It is commonly reported that patients with PCOS are obese and/or hyperinsulinemia besides the signs and symptoms of Rotterdam’s criteria. A case-control study was conducted categorizing PCOS patients based on thyroid-stimulating hormone (TSH) levels, body mass index (BMI), which showed a significant correlation with insulin secretion, insulin resistance, dehydroepiandrosterone sulfate (DHEA), and cortisol levels in obese women with PCOS. In another group of non-obese PCOS patients, TSH levels positively correlate with only waist-to-hip ratios [15]. The excess body weight appeared to promote the interaction between hormonal and metabolic imbalances of PCOS with the presence of SCH [15]. Another study was done in India also noted a significantly higher prevalence of PCOS in women with euthyroid CLT. These women were also noted to have a higher BMI, waist circumference, and systolic blood pressure [16]. Researchers have suggested that due to the altered environment of the body caused by obesity, there is an elevation of pro-inflammatory markers and insulin resistance. This appeared to result in a relative T3 deficiency and an increase in TSH levels due to decreased deiodinase-2 activity at the level of pituitary through undetermined processes [17]. Leptin, a hormone formed primarily in adipose cells, helps regulate energy by inhibiting or stimulating hunger, via the hypothalamus in obese patients, has also been theorized to result in increased thyroid releasing hormone (TRH) secretion [18]. Thus, it may seem that excess amounts of visceral adipose tissue may be attributed as an indicator of TSH concentration in obese patients [17]. SCH patients have also been noted to have a higher presence of low-density lipoprotein levels with no changes in other lipid profile parameters, or insulin resistance [19]. Thus, this could indicate a precursor to the development of thyroid disorders. As data has confirmed TSH strongly correlates with higher low-density lipoprotein (LDL) concentrations in PCOS patients [20]. 

Hormonal Factors

**Figure 1:**
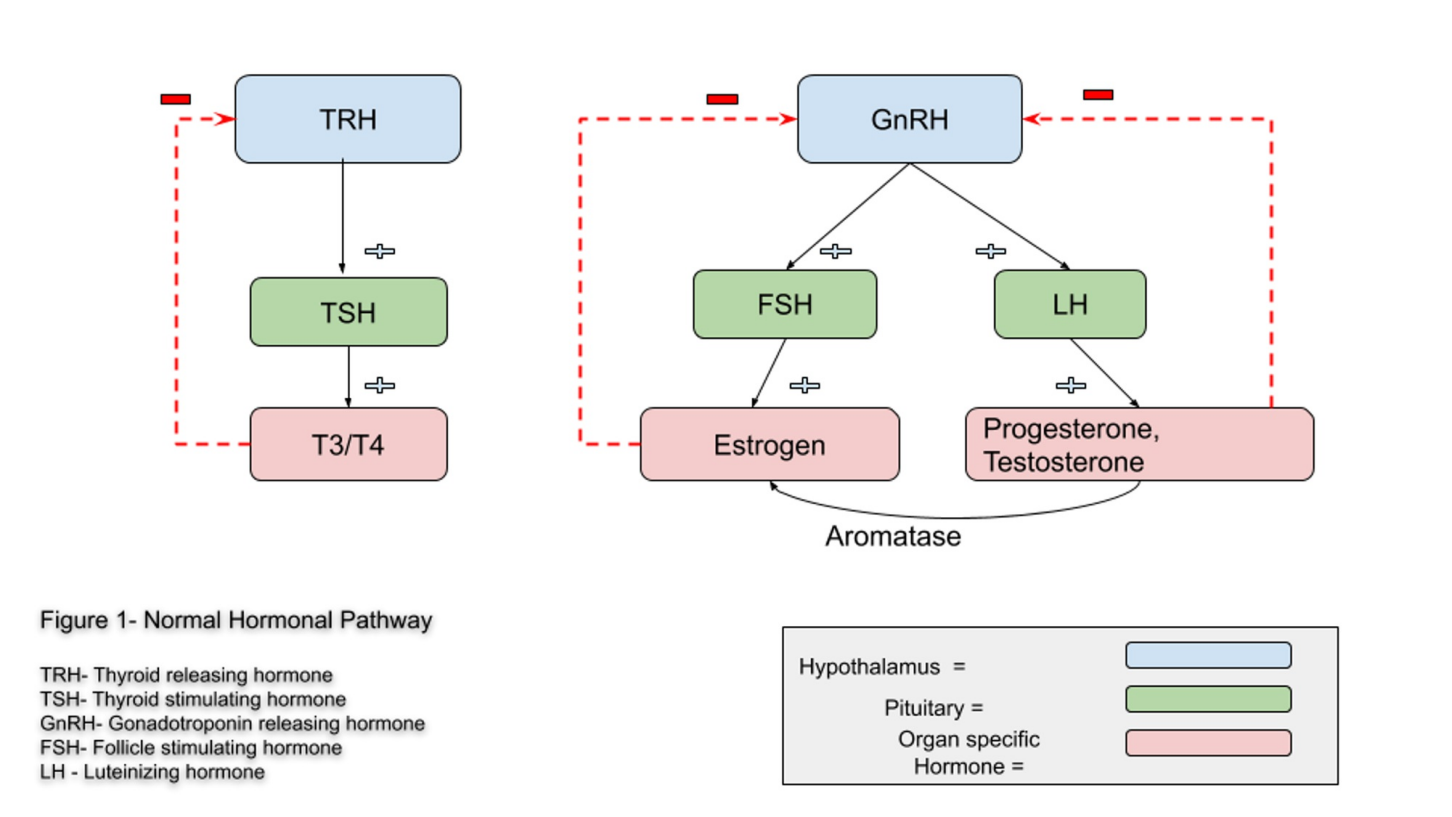
Normal hormonal pathway TRH: thyroid releasing hormone; TSH: thyroid stimulating hormone; GnRH: gonadotropin-releasing hormone; FSH: follicle stimulating hormone; LH: luteinizing hormone

PCOS has also been known to cause hormonal dysfunctions from the low levels of progesterone unable to suppress at the hypothalamic level, leading to an increase in gonadotropin-releasing hormone (GnRH) and luteinizing hormone (LH) pulse frequencies (Figure 1). The resultant effects seen of this hormonal imbalance are hyperandrogenism, which can also be converted to estrogen by aromatase leading tohyperestrogenism via the normal hormonal system. Research has also shown inflammatory and/or immune reactions to speed-up the process of peripheral conversion of androgen precursors to estrogen, mediated by aromatase [21]. Thus, confirming studies which have proposed excess estrogen as an association with an increased incidence of various autoimmune diseases in females rather than males. Estrogen receptors seem to have a proliferative action on B-lymphocytes as well as on T-cells and macrophages [18]. A cohort study done in Germany on PCOS patients revealed the contribution of increased estrogen to progesterone ratio to a threefold higher prevalence of AIT as well as an indication of early manifestation of the disease [8]. Multiple trials done in PCOS patients have shown a higher presence of thyroid-related antibodies such as anti-TSH, anti-TPO, and anti-Tg levels [15]. Thus the increases of thyroid autoantibodies seen in PCOS patients were attributed to the increase in estrogen and estrogen/progesterone ratios.

Immunologic Factors

Much research also indicates an increased prevalence in a variety of autoimmune conditions in women with PCOS and other various causes of unexplained infertility [22]. Autoimmune diseases affect approximately 8% of the population, of which 78% are women [23]. Sex hormones seem to have a connection with the immune response to infections in susceptible persons, leading to an increased prevalence of autoimmune diseases in women rather than men [23]. Various autoimmune antibodies were seen to be significantly increased particularly in PCOS patients. Antibodies seen on the rise were anti-nuclear antibody (ANA), anti-smooth muscle, anti-histone, anti-double-stranded DNA (dsDNA) [24,25]. Many of the studies and trials found via this literature search also coincided with immune-related factors such as increases in serum TSH, antibodies such as anti-TPO, anti-TG [26,27]. As seen numerous studies have revealed a larger increase in thyroid-related factors rather than non-specific autoimmune factors [26,27]. Thus, this data concludes that the increase in thyroid autoantibodies should be taken as a pragmatic approach for further research, in elucidating a distinct cause, by an autoimmune process. These immunologic processes may lead us to the solution or an approach in preventing patients at risk of acquiring thyroiditis.

Genetic Factors 

The prevalence of both thyroid and PCOS individually is seen to be prevalent in more than 70% of families and twins [28]. A few possible common genetic backgrounds have been discovered, which may be the key to linking both conditions. Some studies have shown polymorphisms of the PCOS related gene for fibrillin 3 (FBN3), gonadotropin-releasing hormone receptor (GnRHR) and CYP1B1 encoded to function for estradiol hydroxylation, all of which may be involved in the pathogenesis of HT and PCOS [29]. Fibrillins mediate the action of transforming growth factor-beta (TGF-β), which is a key player in immune tolerance as it stimulates regulatory T cells (Tregs), which function to inhibit the excessive immune response. While levels of TGF-β1 were confirmed to be found lower in HT as well as in PCOS women carrying allele 8 of D19S884 in the FBN3 gene, vitamin D deficiency was also seen to be decreased along with decreased Tregs. When lower levels of TGF-β and Tregs are seen, it may coincide with uninhibited autoimmune processes resulting in a higher incidence of HT and assumed in PCOS [28]. With respect to this data found, very minimal studies have been conducted in appreciating the given genetic polymorphisms, additional studies should be conducted to further strengthen this data, as it is commonly prevalent in families (Table 1).

**Table 1:** Summary of studies associating factors

AUTHOR/YEAR	STUDY TYPE/ LOCATION	SAMPLE SIZE	FACTOR	RESULTS	CONCLUSION
Janssen et al. 2004 [8]	Clinical Trial at the University of Munich, Germany	343	Hormonal	PCOS patients were characterized by an increased LH/FSH ratio, low progesterone, elevated testosterone and a high prevalence of hirsutism (PCOS 83%, control 3%; mean hirsutism score 12+/-5 and 3+/-2 respectively), but no differences in estrogen levels were found. Thyroid function and thyroid-specific antibody tests revealed elevated thyroperoxidase (TPO) or thyroglobulin (TG) antibodies in 14 of 168 controls (8.3%), and in 47 of 175 patients with PCOS (26.9%; P<0.001)	This prospective study demonstrates a threefold higher prevalence of AIT in patients with PCOS, correlated in part with an increased estrogen-to-progesterone ratio and characterized by the early manifestation of the disease.
Tagliaferri et al. 2016 [15]	Clinical Trial at Università Cattolica Sacro Cuore, Rome.	242	Metabolic	The condition of subclinical hypothyroidism was found in 14.28% of PCOS women vs 1.14 % of controls (P	When PCOS patients were classified on the basis of BMI, TSH levels significantly correlated with insulin secretion, insulin resistance, DHEAS, and cortisol levels in obese PCOS women. In nonobese PCOS patients, only waist-to-hip ratio values were correlated with TSH-----The presence of SCH is associated with endocrine and metabolic imbalances of PCOS, and the excessive body weight seems to promote this interplay.
Hefler-Frischmuth et al. 2010 [24]	Clinical Trial at Medical University of Vienna	218	Immunology	Women with PCOS had significantly elevated serum levels of antihistone and anti-dsDNA antibodies, whereas serum levels of ANAs and antinucleosome antibodies were similar between the two groups. When serum levels of ANAs, antihistone, antinucleosomes, and anti-dsDNA antibodies were correlated with clinical and biochemical parameters, a significant correlation between serum levels of ANAs and serum TSH was established.	Study shows that serologic parameters of autoimmunity (i.e., antihistone and anti-dsDNA antibodies) are elevated in women with PCOS. The role of autoimmunologic processes in PCOS can be suspected.
Gaberšček et al. 2015 [28]	Systematic literature search of articles published until September 2013.	343	Genetic	Among them, the FBN3 gene variants seem to be the most plausible candidates due to their influence on TGFβ activity, and consequently, on the level of Tregs. After all, the role of TGFβ has already been implicated in the pathogenesis of PCOS and HT. Additionally, vitamin D deficiency was shown to decrease Tregs.	There is no doubt that genetic susceptibility contributes to the development of both disorders in more than 70% as shown by family and twin studies. Until now, a common genetic background has not yet been established. However, several gene polymorphisms associated with PCOS could also influence HT incidence.
Petrikova et al. 2015 [27]	Clinical Trial at University Hospital of Louis Pasteur, Kosice, Slovak Republic.	360	Immunology	There were no significant differences in the prevalence of ANA, SSA, SSB, anti-dsDNA, anti-RNP, ANCA/MPO or ANCA/PR3 between PCOS and controls. The prevalence of ACLA IgG was higher in controls than PCOS (5.4% v.s. 0%, P=0.011). Patients had a higher prevalence of anti-TPO antibodies (18.75% v.s. 7.35%, P=0.045) and slightly but not significantly higher prevalence of autoimmune thyroiditis (18.75% v.s. 10.29%) than controls.	The prevalence of non organ-specific autoantibodies in PCOS women is low and similar to controls. On the other hand, we found a slightly higher prevalence of thyroid autoimmunity in PCOS women

Recommendations

This data concludes that they should take the gene markers and increase in thyroid autoantibodies as a pragmatic approach in further research to elucidate a distinct caused by an autoimmune process. Additionally, hormonal and metabolic factors should not be overlooked. These factors should be taken into consideration as markers of future incidence of disease in PCOS patients and for preventative measures. We should also consider further research in genetic markers, as these markers may further explain precursors of alternative immunologic diseases. From the research seen in this study, we should also consider retrospective cohort studies in thyroid patients. Following thyroid patients may also reveal the risk of PCOS incidence and other possible autoimmune diseases. From further research, we may also learn additional preventative measures such as screening or markers that may also need to be examined in the commonly seen population of thyroid patients. With immune disorders commonly seen, we should also consider a new perspective such as environmental factors that may include foods and lifestyle. As the world around us is changing, we should consider the changes that may also affect our current and future health. 

## Conclusions

The purpose of this review was to find a correlation between PCOS and thyroiditis. The high-risk correlation of thyroid disease in PCOS patients is highly undeniable as shown in the numerous trials and research conducted globally. Many of the factors discussed seem to all contribute in their own manner to increasing the risk of thyroiditis. The most distinct and agreed upon factor by most studies and trials found via this literature search support the notion of an immunologic cause. We commonly see this cause in women, most likely attributed to their hormonal components. However, genetic studies also seem to be inter-related to immunologic processes as a potential source of induction. Over the years, as humans have also evolved metabolically and environmentally, it seems to have also taken a toll on our bodies' immunologically. As some of these factors may be modifiable, there is yet much more in-depth studies necessary for comprehensive knowledge of these associations for future prevention and treatment.
